# Cold Atmospheric Plasma Is a Potent Tool to Improve Chemotherapy in Melanoma In Vitro and In Vivo †

**DOI:** 10.3390/biom10071011

**Published:** 2020-07-08

**Authors:** Mina Alimohammadi, Monireh Golpour, Farshad Sohbatzadeh, Seyedehniaz Hadavi, Sander Bekeschus, Haleh Akhavan Niaki, Reza Valadan, Alireza Rafiei

**Affiliations:** 1 Department of Immunology, Molecular and Cell Biology Research Center, School of Medicine, Mazandaran University of Medical Sciences, Sari 4847191971, Iran; mina.alimohammadi1369@gmail.com (M.A.); valadan.reza@gmail.com (R.V.); 2 Molecular and Cell Biology Research Center, Student Research Committee, Faculty of Medicine, Mazandaran University of Medical Science, Sari 4847191971, Iran; 178greenbio@gmail.com; 3 Department of Atomic and Molecular Physics, Faculty of Basic Sciences, University of Mazandaran, Babolsar 4741613534, Iran; f.sohbat@umz.ac.ir (F.S.); niaz.hadavi@gmail.com (S.H.); 4 ZIK Plasmatis, Leibniz Institute for Plasma Science and Technology (INP), 17489 Greifswald, Germany; sander.bekeschus@inp-greifswald.de; 5 Cellular and Molecular Biology Research Center, Health Research Institute, Babol University of Medical Sciences, Babol 4817813748, Iran; halehakhavan@yahoo.com

**Keywords:** apoptosis, B16F10, combination therapy, dacarbazine, plasma medicine

## Abstract

Malignant melanoma is a devastating disease. Because of its aggressiveness, it also serves as a model tumor for investigating novel therapeutic avenues. In recent years, scientific evidence has shown that cold atmospheric plasma (CAP) might be a promising modality in cancer therapy. In this study, we aimed to evaluate the effect of CAP generated by an argon plasma jet alone or in combination with dacarbazine (DAC) on melanoma cells in vitro and in vivo. The effects of the CAP on inducing lipid peroxidation and nitric oxide production were higher in B16 melanoma cells in comparison to non-malignant L929 cells. Assays on cell growth, apoptosis, and expression of genes related to, e.g., autophagic processes, showed CAP to have a substantial impact in melanoma cells while there were only minoreffects in L929 cells. In vivo, both CAP monotherapy and combination with DAC significantly decreased tumor growth. These results suggest that CAP not only selectively induces cell death in melanoma but also holds promises in combination with chemotherapy that might lead to improved tumor control.

## 1. Introduction

Melanoma skin cancer has a high mortality, and its incidence is increasing in recent decades [1]. Melanoma originates from skin-resident melanocytes and is an aggressive type of cancer that accounts for more than 75% of skin cancer mortalities [2]. In the advanced stages of melanomas, surgical resection, systemic therapy, and adjuvant cytotoxic chemotherapy show limited efficacy, leading to a five-year survival rate of only 20% [3]. Resistance to anticancer therapy is a common clinical problem in many cancer patients. Resistance can be attributed to a range of mechanisms involved, for instance, tumor heterogeneity, metabolism, stress, tumor microenvironment, and genetic or epigenetic changes that are associated with remedy failure [4].

Melanoma cell lines can be resistant to apoptosis and targeted therapy in preclinical studies [5]. Autophagy is discussed as a potential mechanism of this finding [6,7]. Autophagy is generally a double-edged sword. On the one hand, autophagy can protect cells from cytotoxic agents and cause cancer cells to survive. On the other hand, it can lead to type II cell (autophagy-induced) cell death [8]. This suggests that autophagic cell death could be established as a caspase-independent alternative cell death mechanism [9]. Therefore, autophagy might be a useful and novel therapeutic target for the destruction of cancer cells, especially in metastatic melanoma and drug-resistant cancers, in which apoptosis is impaired [6,10,11]. Autophagy is a highly regulated cellular catabolic process that protects cells from unfavorable protein aggregation and damaged organelles and enhances cellular homeostasis, immunity, tumor suppression, genome stability, metabolism, and longevity [12,13,14]. In response to stressors such as hypoxia or starvation, the initiation of autophagy is mediated by many complex proteins such as Unc-51-like autophagy activating kinase 1(ULK1), class III phosphoinositide 3-kinase (PI3K), ATG14L, VPS15, beclin1, autophagy-related gene 5 (ATG5), and microtubule-associated protein light chain 3 (LC3) [15].

In the last decade, cold physical atmospheric plasma (also termed cold atmospheric pressure plasma, CAP) has attracted the attention of many scientists because it has been recognized as a potentially useful tool for the treatment of many types of cancer, including melanoma [16,17,18]. CAP is a partially-ionized gas at atmospheric pressure containing electrons, positive and negative ions, reactive oxygen species (ROS), and nitrogen (RNS). Cold physical plasma devices generate plasmas at low temperatures in the range of the body temperature and hence avoid causing thermal harm to cells or tissues [19,20,21]. Several CAP devices have been used in preclinical anticancer studies. The plasma sources differ in, e.g., geometrical configuration, driving frequency, high voltage waveforms, and working gas. Several types are known, for instance, the floating-electrode dielectric barrier discharge (DBD) [22], the surface micro discharge (SMD) [23], and plasma jets [24].

It has been reported that CAP induces oxidative stress in various types of cancer cells and leads to an increase of apoptosis [25,26,27] and other cell death pathways [28,29,30]. Moreover, studies that investigated the potential of CAP treatment to be combined with traditional chemotherapy are scarce. In this study, an argon plasma jet was used to tackle melanoma in vitro and in vivo. It was demonstrated the plasma treatment showed selective cytotoxic effects, and that this potential was enhanced when combined with dacarbazine chemotherapy in terms of tumor regression.

## 2. Materials and Methods

### 2.1. Cold Atmospheric Pressure Argon Plasma Jet

**Figure 1:**
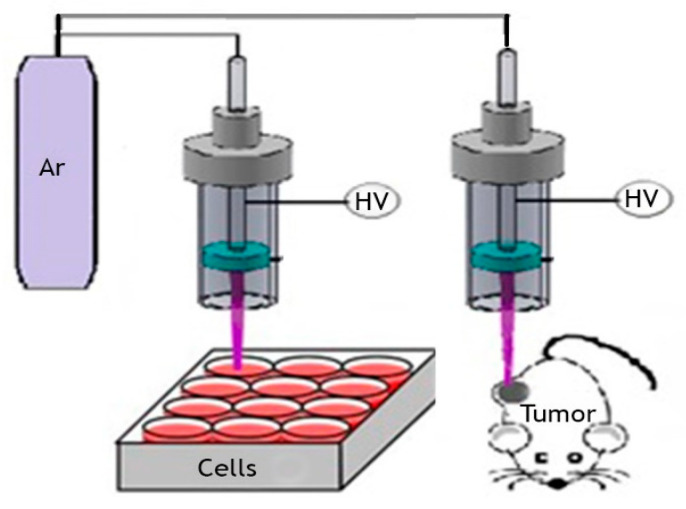
Schematic of the argon-seeded plasma jet applied in vitro and in vivo.

An argon plasma jet was used as described before [31]. Briefly, in our designed device, the distance between the two electrodes is ~7 mm. In pure argon gas (2.5 L/min) through a quartz tube, a plasma discharge between the two electrodes is generated by using high voltage. The plasma jet’s length is 1.5 cm, with a final diameter of about 0.3 cm and gas temperature of 35 to 40 °C. The discharge current between the metal wire and the ring electrode is 10 mA (Figure 1). In this device, the relative emission intensity of the hydroxyl radical (in 308 nm) is the largest emission line among the other detected plasma species. Other important chemical reactive plasma species generated with the device are O, $O_{2}^{+}$, $O_{4}^{+}$, $O_{2}^{-}$, $O^{-}$, $O_{3}$, NO, singlet oxygen molecule $O_{2}\left(a^{1}\Delta_{g}\right)$, and H_2_O_2_.

### 2.2. Cell Culture

The malignant murine melanoma cell line (B16F10) and the non-malignant murine cell line (L929) were purchased from the Pasteur Institute (Tehran, Iran). The cells were cultured in Roswell Park Memorial Institute 1640 (RPMI-1640) medium (Attocell, Austria) supplemented with 10% (*v*/*v*) fetal bovine serum (FBS), 100 U/mL penicillin, and 100 µg/mL streptomycin under standard culture conditions (37 °C, 5% CO_2_, and 95% humidity).

### 2.3. Metabolic Activity, Cell Viability, and Apoptosis

The MTT (3-(4, 5-dimethylthiazol-2-yl)-2, 5-diphenyltetrazolium bromide) assay is frequently used to measure the cytotoxic effects of different agents in cells that compromise their metabolic activity. A total of 7 × 10^3^ B16 or L929 cells per well were cultured in a 96-well plate with three technical replicates for each group and allowed to grow for 24 h before treatment. We cultivated four groups for each cell line: group 1, untreated cells as a negative control; group 2, Dacarbazine (DAC) (100 μg/mL) treated cells, an alkylating anticancer agent routinely used for the treatment of metastatic melanoma; group 3, H_2_O_2_ (0.1 mM) treated cells as a control for oxidative stress; group 4, CAP treatment for 45s. Twenty-four hours after the treatment, the culture medium was removed, and the cells were washed with PBS before 50 µL of MTT (3-(4, 5-dimethylthiazol-2-yl)-2, 5-diphenyltetrazolium bromide, Sigma, USA) solution (5 mg/mL in PBS) was added to each well. After 4 h of incubation at standard conditions, formazan crystals were solubilized by the addition of 150 µL of dimethylsulfoxide (Merck, Darmstadt, Germany) to each well. The absorbance was measured at 570 nm using a BioTek microplate reader (BioTek, Winooski, VT, USA).

To analyze cell viability, the fluorescent dye acridine orange (AO) was used. The dye passes through the cell membrane and binds to DNA. Hence, all cells stain positive for this dye when using fluorescence microscopy. By contrast, ethidium bromide (EB) is unable to pass through cell membranes of live cells, but it can enter dead cells where it then binds to DNA to mark cells with compromised cell membranes (terminally dead cells). For AO/EB staining, 7 × 10^3^ cells/well were cultured in 96-well plates. After treatment and overnight incubation, 25 µL of PBS containing 2 µL of AO/EB mixture (100 µg/mL) was added to each well. Fluorescence microscopy was performed with four to six random fields of view for each well using an inverted fluorescent microscope at 400× magnification.

To determine apoptosis, the Annexin V Apoptosis Detection Kit (BD Biosciences, Franklin Lakes, NJ, USA) was used. Briefly, after 24 h of culture, the cells were washed with PBS and resuspended in 1× binding buffer before labeling with Annexin V-FITC and propidium iodide (PI, 1 mg/mL). Samples were mixed gently and incubated at room temperature in the dark for 15 min. After washing in binding buffer, cells were analyzed using flow cytometry (Partec, Münster, Germany).

### 2.4. Nitric Oxide (NO) and LipidPeroxidation

To quantify nitrite, a product of NO, the Griess assay was employed [32]. Briefly, the standard solution was prepared using ionized water and concentrations of 0, 5, 10, 15, 20, and 25 μM ofNaNO_2_. Each sample was added in five technical replicates per 96-well microplates. In each well, 100 μL of each sample and the standard mentioned above were added separately. Then, 50 μL of 2% sulfanilamide (in 5% HCl) and 50 µmL of 0.1% N-naphthyl-ethylenediamine were added to each well and incubated for 30 min at 37 °C. After incubation, the optical density of each reaction mixture was read at 540 nm using a BioTek microplate reader (BioTek, Winooski, VT, USA). Linear regression analysis of the standard samples was performed for quantification of the nitrite concentrations in µg/mL.

Lipid peroxidation was determined by measuring the levels of malondialdehyde (MDA) (28). Briefly, after incubation of the cells for 24 h, the supernatant was collected and shaken in the presence of 2.5 mL of 20% trichloroacetic acid (TCA) in a 10 mL centrifuge tube to precipitate protein. One milliliter of 0.6% TBA was added to the mixture, shaken, and heated for 20 min at 90 °C in a water bath. The precipitate was pelleted by centrifugation at 4000× *g* for 15 min yielding a chromogenic product. MDA levels were read at 535 nm (BioTek, Winooski, VT, USA).

### 2.5. Animal Experiment

Six- to eight-week-old male BALB/c mice were housed under standard conditions at the Institute of Laboratory Animals of Mazandaran University of Medical Sciences. The protocol of this study was approved by the Ethical Committee of Medical Researches of Mazandaran University of Medical Sciences. To grow melanoma tumors, 5 × 10^5^ B16 cells in 100 μL PBS were injected subcutaneously into the right flank of mice. Ten to twelve days after B16F10 tumor cell inoculation, the tumor diameter reached 5 mm. Tumor-bearing mice were randomly divided into four groups, including (a) untreated controls, (b) chemotherapy using DAC, (c) plasma jet treatment (CAP), and (d) combination therapy using DAC and CAP. DAC was injected intravenously at a dose of 0.1 mg/kg for five days. CAP treatment was performed for 5 min three times with a five-day interval. The plasma jet nozzle was set up on the top of the tumor mass at a distance of 15 mm to the skin surface and was continuously moved across the tumor surface during treatment. The tumor size was measured using a digital caliper and calculated using the following formula: π/6 × length × width × width. For tumor tissue analysis, mice were sacrificed, and the tissue was stored at −70 °C until RNA extraction.

### 2.6. Histology

Melanoma samples were fixed in 10% buffered formalin and embedded in paraffin using the classical histological method. Three-micrometer thick sections were cut using a Rotary Leica microtome (Leica Biosystems, Nussloch, Germany) and attached to microscopy slides. For diagnostic purposes, the slides were stained with hematoxylin and eosin (H&E) and imaged using brightfield microscopy.

### 2.7. RNA Quantification

Total RNA was extracted from 1 × 10^6^ B16 or L929 cells treated in vitro, and from 50 mg of tumor tissue samples from each mouse group at different time points of examination, using an RNA extraction kit (FervorGen, Taiwan) according to the manufacturer’s instructions. The quantity and quality of the extracted RNA were measured based on the absorbance ratio (A260/A280) and agarose gel electrophoresis, respectively. The total RNA was stored at −70 °C until used for further assays. The expression of Bax, Bcl2, Caspase 3, LC3, and ATG5 mRNA levels were quantified by stem-loop Taqman real-time PCR assay, using a unique sequence index (USI) barcode and probe described by Fattahi et al. [33,34]. GAPDH mRNA was used as an endogenous housekeeping control for all of the genes. All experiments were carried out in triplicate. Primers were designed using AlleleID 6.0 software (Table 1). For analysis of the in vitro samples, total RNA was extracted from each sample and transcribed into cDNA using mRNA specific USI RT-PCR primers and Reverse Transcriptase (GeNet Bio, Chungcheongnam-do, Korea). Gene expression analysis was performed using the Step One real-time PCR device (Applied Biosystems, Foster City, CA, USA) and HotStarTaqPlus DNA Polymerase (QIAGEN, Hilden, Germany) was used for the real-time PCR reaction. Amplification was performed according to Fattahi et al. [33,34]. RNA levels (relative fold change) were determined using the Livake method [35].

### 2.8. Statistical Analysis

Quantitative variables were shown as mean + or ± standard deviation (SD). Statistical analysis was determined for differences between groups with normal distribution by one/two way of analysis of variance (ANOVA). Tukey’s post hoc test was used to perform pairwise comparisons between groups. In all comparisons, *p*-values <0.05 were considered as statistically significant differences.

## 3. Results

### 3.1. CAP Selectively Induced Cellular Damage and Oxidation in B16 Cells

**Figure 2:**
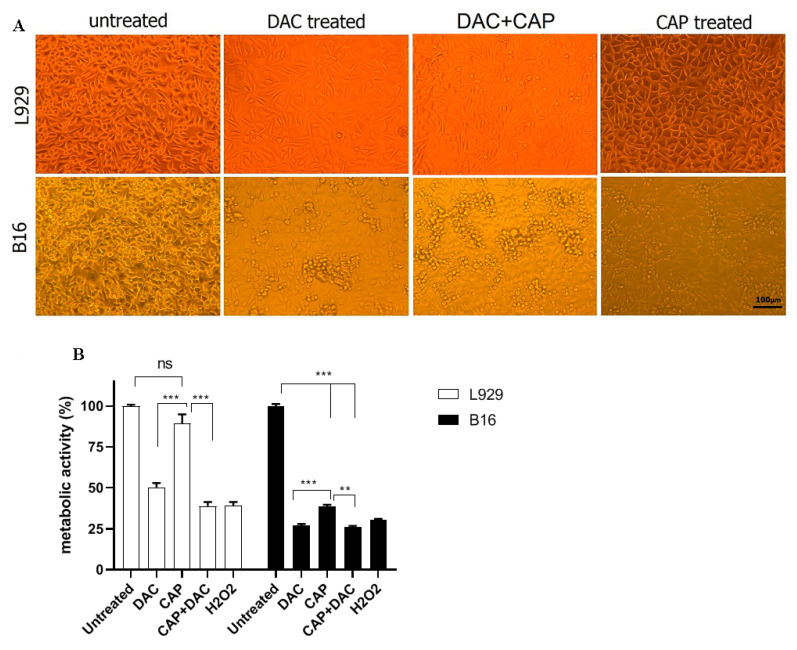
The effect of CAP, DAC, or combined treatment on cell morphology and metabolic activity of B16 and L929 cells.The cells were treated with CAP for 45 s, DAC (100 μg/mL), CAP+DAC combination, or were left untreated, and evaluated after 24 h. (**A**) Metabolic activity was assessed at 24 h post-treatment using the MTT assay. (**B**) Since ROS production is a well-defined mechanism of plasma, hydrogen peroxide was used as a positive control. The results are representative of mean +SD of three independent experiments. Metabolic activity was calculated by the below formula: (OD_570nm_ of treated cells/OD_570nm_ of untreated cells) × 100. The scale bar is 100 µm. NS means not significant, and *** and ** represent *p* < 0.001 and *p* < 0.01, respectively.

**Figure 3:**
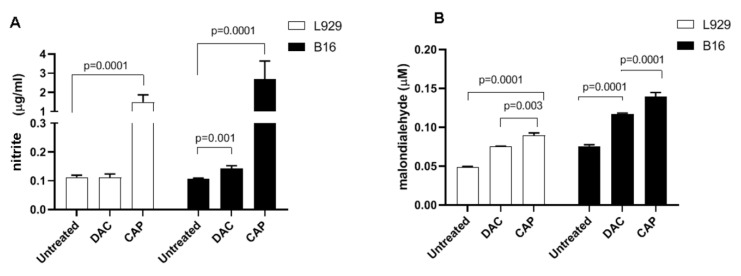
Quantification of nitrite and MDA. The levels of nitrite (**A**) and MDA (**B**) were measured in B16 and L929 cell supernatants and following exposure to the mono and combination treatment. Data are presented as the mean + SD of five technical replicates experiments.

Cell lines of malignant, murine melanoma cells (B16) and non-malignant murine fibroblasts (L929) were used in this study. CAP and DAC treatment led to morphological changes indicative of cell damage (Figure 2A). For CAP, this was more pronounced in B16 over L929 cells, while DAC effects appeared to be similar in both cell types. Quantitative analysis of metabolic activity was performed using the MTT assay. CAP-treatment induced a significant reduction in metabolically active B16 cells compared to untreated B16 controls (Figure 2B). Again, the effects were less pronounced in L929 cells, while DAC was similarly toxic in both cell lines. To understand the contribution of endogenous (or plasma-derived) RNS such as NO in the toxicity, nitrite-a product of NO- was quantified in the cell culture supernatants. CAP treatment significantly increased NO production in both L929 and B16 cells compared with the untreated control (Figure 3A). The effect of DAC was minor in both cell lines, while the effect of CAP was stronger in B16 compared to L929 cells. Both RNS and ROS can contribute to lipid peroxidation and oxidant-induced cell death. To this end, malondialdehyde (MDA), an indicator of lipid peroxidation, was quantified. Both plasma and DAC treatment resulted in elevated MDA levels within B16, as well as L929 cells (Figure 3B). Altogether, plasma (selectively) and DAC (non-selectively) treatment led to morphological changes and decreased metabolic activity in the tumor cells, while elevated RNS lipid peroxidation levels were observed in both B16 and L929 cells to a similar extent with plasma treatment.

### 3.2. CAP Selectively Induced Apoptosis in B16 Melanoma Cells

**Figure 4:**
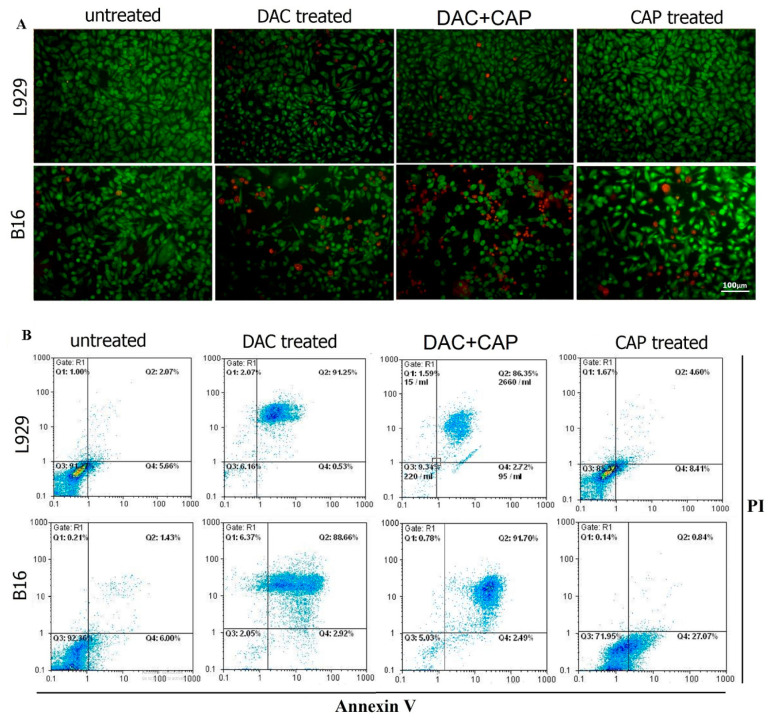
Effect of CAP and DAC on apoptosis.The pattern of AO/EB staining in B16 tumor cells or non-malignant L929 cells treated with CAP (45-sec exposure) or DAC (100 µg/mL), or without any treatment (untreated). (**A**) Green fluorescence shows the live cells, while orange fluorescence represents the dead cells. Annexin V/PI flow cytometry analysis of the B16 and L929 cells 24 h post the different treatment regimens. The data represent the percentage of live cells (Q3), early (Q4) or late (Q2) apoptotic cells, and necrotic cells (Q1). (**B**) Data are representative of three independent experiments. The scale bar is 100 µm.

**Figure 5:**
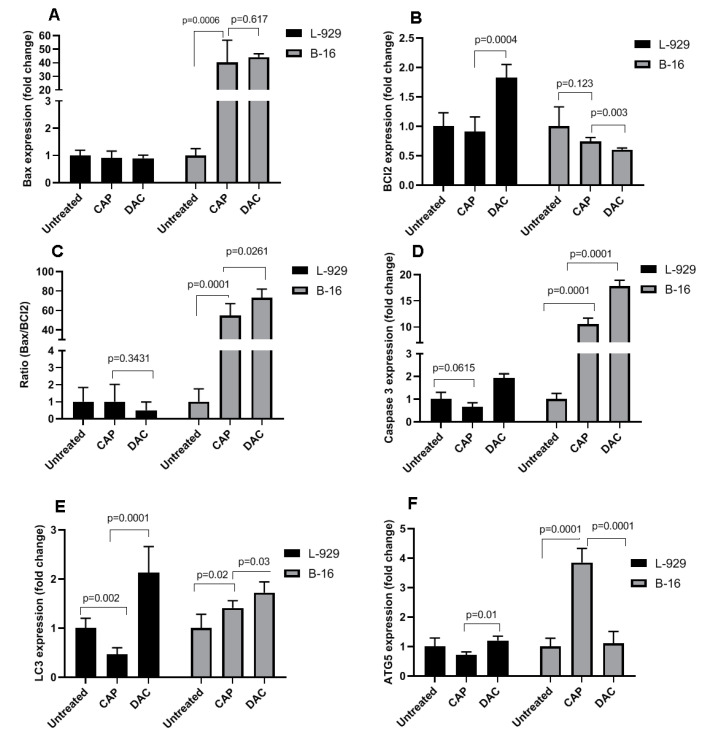
Effect of CAP on apoptotic gene expression in B16 tumor cells and non-malignant L929 cells. (**A**–**F**) 24 h after CAP or DAC treatment, mRNA was extracted, and the expression of apoptotic (Bax, (**A**); Bcl2, (**B**); caspase 3, (**C**); and Bax to Bcl2 ratio, (**D**)) and autophagy-related (ATG5, (**E**); and LC3, (**F**)) genes was evaluated using real-time PCR. In all experiments, GAPDH was considered as the housekeeping control gene. Altered gene expression is shown as fold changes referring to untreated control cells. The results are representative of three independent experiments and show mean + SD.

To further evaluate the effects of CAP on the cell death pattern and apoptosis in B16 and L929 cells, AO/EB fluorescence staining and the Annexin-V/PI assay were employed. AO diffuses into all cells (green), while EB only diffuses across the membrane of dead cells (red). The AO/EB staining revealed a higher occurrence of cell death in CAP-treated B16 cells compared with controls (Figure 4A). On the contrary to the impact of DAC in non-malignant L929 cells, CAP treatment had no significant toxic effect in L929 cells. To further confirm the effect of CAP on tumor cells, apoptosis analysis was performed, and representative flow cytometry dot plots of Annexin V and PI are shown (Figure 4B). The percentage of apoptosis was significantly higher in B16 cells after CAP treatment compared with controls. Meanwhile, flow cytometry revealed that DAC induced high apoptosis rates in both B16 tumor cells and non-malignant L929 cells. To analyze apoptosis on the molecular level, the expression of apoptotic genes, including Bax, Bcl2, and caspase 3 was evaluated in B16 and L929 cells and using stem-loop Taqman real-time PCR assay (Figure 5). The expression of Bax and caspase 3 but not Bcl2 was significantly increased after CAP treatment in B16 tumor cells in comparison to untreated controls (Figure 5A,B). The Bax/Bcl2 ratio was significantly changed in B16 tumor cells but not in non-malignant L929 cells following Ar-plasma exposure (Figure 5C). These modulations were in accordance with changes in caspase 3 expression (Figure 5D). In addition, CAP significantly increased the mRNA expression of LC3 and ATG5 in B16 tumor cells in comparison to untreated control cells (Figure 5E,F). Both genes are associated with autophagic processes. A similar result was observed for DAC in B16 cells for LC3. In L929 cells, however, CAP treatment decreased the expression of LC3 and ATG5, while DAC led to an increase and no change, respectively.

### 3.3. CAP Treatment Induced Apoptosis and Tumor Regression in B16 Melanoma-Bearing Mice

**Figure 6:**
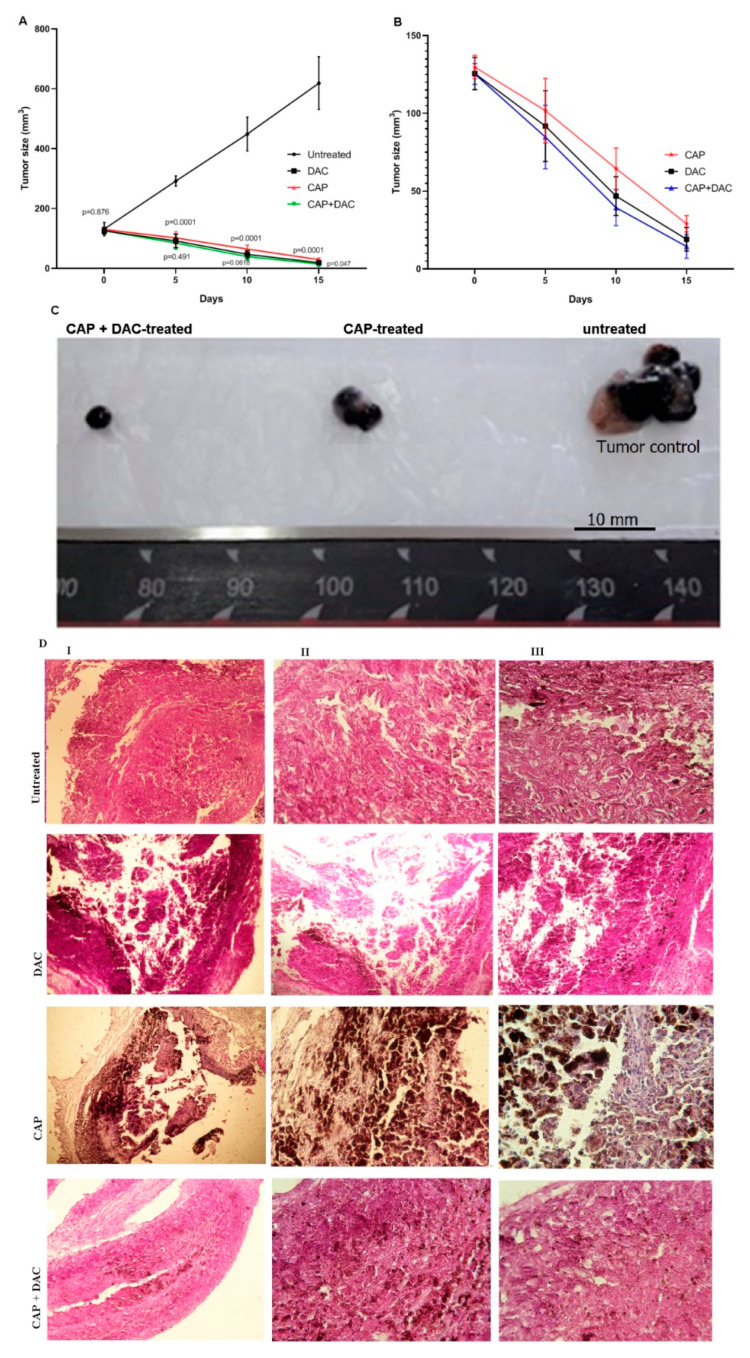
Effect of CAP and DAC mono and combination therapy on melanoma progression in vivo. B16 tumor-bearing mice either left untreated or treated with CAP, DAC, or CAP+DAC combination therapy. Tumor size was evaluated every five days with the statistical comparison of tumor size in the CAP-treated group to the untreated group (upper *p*-values) and between DAC and combination therapy (lower *p*-values). (**A**) Comparison of tumor volume of all groups at a lower scale. (**B**) Representative images of tumors treated with CAP, combination therapy, and of the untreated group (**C**) after two treatments. H&E staining on the tenth day after inoculation of the tumor (**D**) of a nodular tumor that occupied the dermis (I), area of necrosis (II), and pleomorphic and achromic epithelioid malignant melanocytes with vesiculous nuclei and eosinophilic macronuclei (III). Data are presented as mean ±SD of at least five mice for each time point.

To evaluate the in vivo efficacy of the CAP on tumor regression, melanoma-bearing mice were CAP-treated three times in five-day intervals. Tumor progression and regression were followed by measuring tumor size and followed gene expression analysis. In addition, the combined effect of CAP and DAC chemotherapy was also determined in a separate experimental group. There was no significant difference (*p* = 0.876) in tumor size at the start point of intervention (Figure 6A). However, after the beginning of intervention by CAP, DAC chemotherapy, or combination therapy on days 5, 10, and 15, the tumor size was significantly reduced compared to the untreated group from day 5 onwards (Figure 6A,B). Combination therapy seemed to be the most potent modality to regress tumor progression (Figure 6C), although this was not statistically significant. H&E staining additionally confirmed the melanoma nodules in the dermis and their phenotype (Figure 6D).

**Figure 7:**
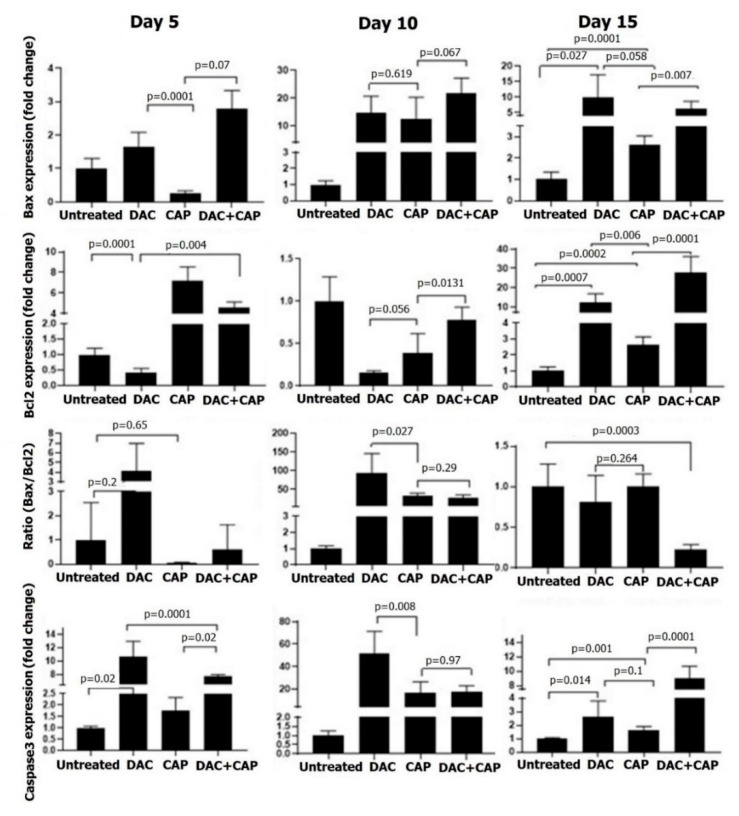
Apoptotic gene expression in melanoma samples subjected to CAP or DAC mono or combination therapy. Shown are results for Bax, Bcl2, and caspase 3 in tumor tissue lysates collected at days 5, 10, and 15. Real-time PCR was performed using GAPDH as a housekeeping control gene. Altered gene expression is shown as fold changes referring to untreated control cells. The results are representative of three mice per time point. Statistical analysis was performed using one-way ANOVA with Tukey’s post hoc test to untreated normalized control.

**Figure 8:**
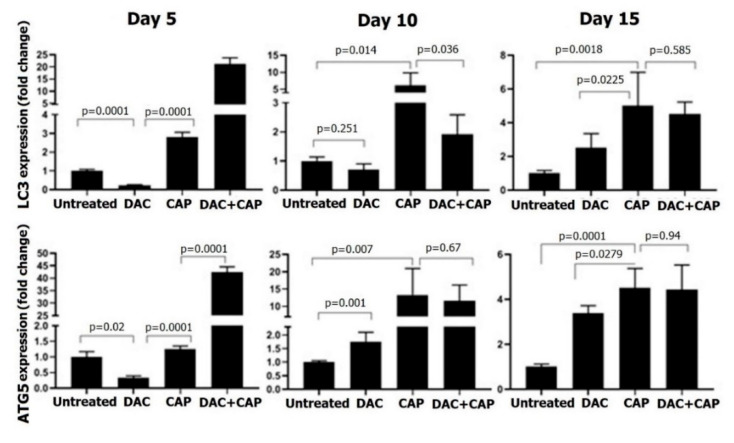
LC3 and ATG5 expression in melanoma samples subjected to CAP or DAC mono or combination therapy. Shown are results for LC3 and ATG5 in tumor tissue lysates collected at days 5, 10, and 15. Real-time PCR was performed using GAPDH as a housekeeping control gene. Altered gene expression is shown as fold changes referring to untreated control cells. The results are representative of three mice per time point. Statistical analysis was performed using one-way ANOVA with Tukey’s post hoc test with the untreated normalized control.

**Figure 9:**
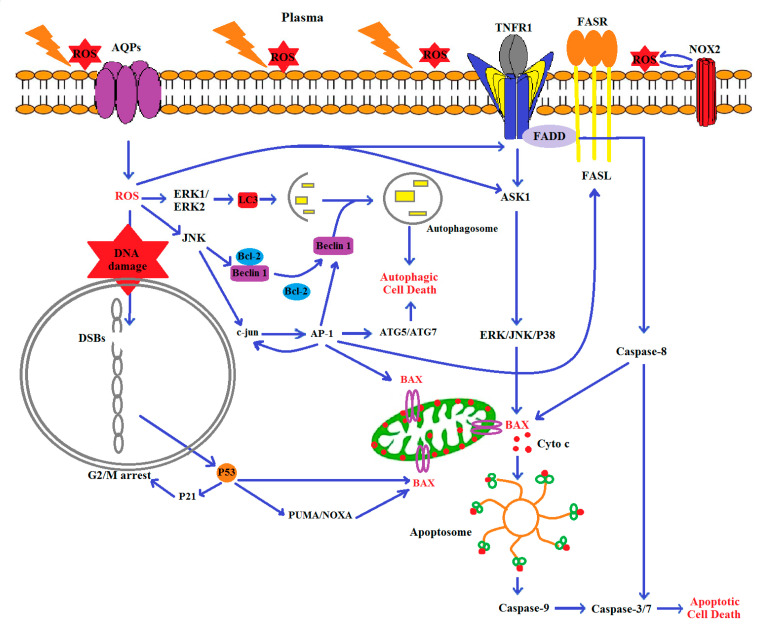
Schematic model of the molecular signal pathway of the proposed cell death crosstalk in B16 cells exposed to plasma induced-ROS. In short, CAP-induced ROS cause oxidative stress. The excessive intracellular ROS can, in turn, activate the TNF-R1 pathway, which leads to ASK1 activation and downstream stimulation of ERK, JNK, or p38. Phosphorylated JNK activates the pro-apoptotic protein, Bax. Bax translocates to the mitochondria and triggers the release of cytochrome C into the cytosol, leading to the activation of caspase-3/7 and eventually causes apoptotic cell death. Intracellular ROS can also suppress cell growth by inducing DNA double-strand breaks (DSBs) and upregulating p53 antitumor activity. P53 arrests the cell cycle via P21 and increases apoptosis based on the mitochondrial pathway by enhancing PUMA/NOXA/Bax. By contrast, ROS can directly stimulate ERK1/ERK2 and JNK. ERK1/ERK2 can induce LC3. JNK phosphorylates Bcl-2 and releases beclin1 associated with LC3 that is involved in autophagic cell death. Alternatively, JNK can induce c-jun phosphorylation. Phospho-c-jun induces AP-1 production, which in turn increases the expression of many genes such as c-jun, beclin 1, Atg5/7, Bax, and FasL. Beclin 1 and Atg 5/7 stimulate autophagic cell death, and FasL can induce Fas receptor (Fas-R) expression and activate caspase 8, which in turn stimulates Bax-oligomerization.

Apoptosis-related genes in tumor tissues were investigated at three different time points of melanoma therapy. On day five, CAP exposure had no significant effect on Bax/Bcl ratio and caspase 3 expressions, while DAC treatment significantly increased the levels of Bcl2 and caspase 3 (Figure 7). With the second dose of the CAP and DAC, as well as the combination treatment on day 10, the Bax/Bcl2 ratio and caspase 3 expression were found to be significantly increased in all conditions. At day 15 in treated mice, changes in the Bax/Bcl2 ratio were abolished, while caspase 3 was significantly elevated, although at a lower magnitude compared to day 10. Compared to mono treatment, the combination therapy gave the most substantial changes on day 15, while it was surprising to find the anti-apoptotic Bcl2 increased the most in this condition. CAP therapy significantly enhanced LC3 and ATG5 expression on day 10 and, to a lesser extent, on day 15 (Figure 8). DAC treatment first led to a significant decline of both markers on day 5, followed by modest changes on day 10, and a stronger increase on day 15. The combination treatment led a very pronounced increase on day 5 with 20-fold (LC3) and 40-fold enhancement in expression of LC3 and ATG5, respectively. On days 10 and 15, LC3 and ATG5 expression were still upregulated with combination therapy but to a lesser extent when compared to day 5. A schematic model of molecular mechanisms of ROS, the main defined CAP-producing substances, on proposed-autophagic/apoptotic cell death crosstalk in B16 melanoma is shown (Figure 9).

## 4. Discussion

Metastatic melanoma is one of the most malignant cancers with high resistance to chemotherapy, radiotherapy, and immunotherapy. Therefore, finding new effective therapeutic modality is essential to induce various cell death processes that would be more difficult to overcome by cancer cells [36]. In this study, we identified CAP as an apoptotic-inducing tool for growth inhibition and tumor regression of B16 melanomas. CAP treatment was selective as it did not affect non-malignant cells to the same extent. In addition, combining CAP treatment with chemotherapy (DAC) increased the potency of the approach. Our results are in line with reports on other tumor cell lines, for instance, melanoma [37,38,39,40,41,42,43], brain cancer [44,45,46], breast cancer [47,48], colorectal cancer [49,50,51,52], lung cancer [53,54,55], leukemia [56,57,58,59,60], liver cancer [61,62,63], and head and neck cancer [64,65,66] that show antitumor effects of plasma treatment, as well as partially also a combination effect with other types of therapies.

CAP generates a plethora of biologically active ROS simultaneously [67,68,69]. These ROS then interact with cell membranes and other compartments, leading to redox signaling and many forms of cell death [70]. Because of metabolic differences between tumor and non-malignant cells, the plasma can selectively act on tumor cells [71]. We found CAP treatment to increase endogenous NO production and lipid peroxidation, both associated with increased cell death in B16 cells. Supraphysiological NO has been previously linked to apoptosis B16 melanoma [72]. Apoptosis can be a significant event in melanoma cells, so the response to cancer treatments in some circumstances depends on the successful induction of apoptotic activity [73]. In this study, AO/EB fluorescence staining and flow cytometry analysis revealed that the CAP and DAC chemotherapy-induced apoptosis in B16 melanoma, while only DAC but not CAP was toxic in non-malignant L929 cells. mRNA expression analysis confirmed this finding. The ratio of Bax/Bcl2 demonstrates the stability and balance between the expression levels of pro and anti-apoptotic genes that play an essential role in the cellular response to stress and exogenous factors resulting in apoptosis [74]. Our data are consistent with other studies that confirmed the increased expression of pro-apoptotic genes in different types of plasma-treated cancer cells [75,76,77,78].

Although there was a weakness in our study as it lacked protein expression data, our data on LC3 and ATG5 gene expression increased with CAP treatment, suggesting the involvement of autophagic processes in B16 cells. Autophagy is mediated by the ULK1 complex. Then the PI3Kcomplex that contains VPS34 (vacuolar protein sorting 34), PI3K, ATG14L, VPS15, and beclin1, helps the organization of the phagophore. Elongation of the phagophore is regulated by the ATG5, ATG12, ATG16 complex, and LC3 [15]. Therefore, ATG5 and LC3 are critical molecules in the induction of autophagic activity. Autophagy is essential for the survival or death of the cells, so that many studies have focused on demonstrating the effect of cancer treatment strategies based on autophagy mechanisms. Although it is well known that autophagy has a significant role in tumor biology [36,79,80], reports focusing on plasma-triggered autophagy induction are lacking. Our results showed that the expression of LC3 and ATG5 mRNA was significantly increased in B16 melanoma cells treated by the CAP, whereas CAP-treated non-malignant L929 cells showed an appreciable reduction in the expression of autophagic-related genes. This might be a useful modality in cancer therapy to specifically overcome resistance to apoptosis-inducing drugs. Autophagy may coincide with apoptosis to induce cell death [81].

The combined effect of the CAP and DAC on melanoma mass regression was more promising than the monotherapy by each one alone. In general, both mono and combination treatment had a remarkable effect on reducing the tumor mass, as seen on day 15. This finding is in accordance with other reports that show the combination of plasma treatment with other conventional therapeutic approaches such as ursolic acid, temozolomide, radiotherapy, or gemcitabine for efficient tumor cell inactivation and removal in vivo [82,83,84]. Our findings of the increased expression of pro-apoptotic genes in tumor tissues in both mono and combination treatment, especially after the second hit at day 10, underline these findings.

## 5. Conclusions

Our findings suggest CAP treatment to be selectively toxic in tumor cells by promoting apoptosis. In addition, adjuvant therapy by CAP might be significantly increased the effectiveness of chemotherapy on tumor cells. Further studies are needed to clarify the signaling pathways involved to better understand plasma therapy as an adjuvant modality in tumor treatment.

## Figures and Tables

**Table 1:** Primer sequences used for stem-loop RT PCR.

Accession Number	Gene Name	Primers 5′ 3′
NM_007527.3	Bax	Specific forward primer: GCGGCTGCTTGTCTGGATC USI RT-PCR primer GTCGTATCCAGTGCTGCGACCGTATGGATGTGTCTGCGGCGTTTTATCATGCACTGGATACGACCGGTGAGGACTC
NM_009741.5	Bcl2	Specific forward primer: CTACGAGTGGGATGCTGGAGATG USI RT-PCR primer GTCGTATCCAGTGCTGCGACCGTATGGATGTGTCTGCGGCGTTTTATCATGCACTGGATACGACGCTGGAAGGAGA
NM_001284409.1	Caspase 3	Specific forward primer: CTCTACAGCACCTGGTTACTATTCC USI RT-PCR primer GTCGTATCCAGTGCTGCGACCGTATGGATGTGTCTGCGGCGTTTTATCATGCACTGGATACGACGTTGCCACCTTC
NM_025735.3	LC3	Specific forward primer CTTCGCCGACCGCTGTAAG USI RT-PCR primer GTCGTATCCAGTGCTGCGACCGTATGGATGTGTCTGCGGCGTTTTATCATGCACTGGATACGACGATCACCGGGAT
NM_053069.6	ATG5	Specific forward primer GTCGCCCCTGAAGATGGAGAG USI RT-PCR primer GTCGTATCCAGTGCTGCGACCGTATGGATGTGTCTGCGGCGTTTTATCATGCACTGGATACGACGCTCAGCCACT
NM_001289726.1	GAPDH	Specific forward primer TTGTCAAGCTCATTTCCTGGTATG USI RT-PCR primer GTCGTATCCAGTGCTGCGACCGTATGGATGTGTCTGCGGCGTTTTATCATGCACTGGATACGACGGAGGCCATGTAG

## References

[1] Rastrelli M., Tropea S., Rossi C.R., Alaibac M. (2014). Melanoma: Epidemiology, risk factors, pathogenesis, diagnosis and classification. Vivo.

[2] Liu X., Wu J., Qin H., Xu J. (2018). The role of autophagy in the resistance to braf inhibition in braf-mutated melanoma. Target. Oncol..

[3] Siegel R., Ma J., Zou Z., Jemal A. (2014). Cancer statistics, 2014. Ca A Cancer J. Clin..

[4] Fecher L.A., Amaravadi R.K., Flaherty K.T. (2008). The mapk pathway in melanoma. Curr. Opin. Oncol..

[5] Hersey P., Zhang X.D. (2008). Adaptation to er stress as a driver of malignancy and resistance to therapy in human melanoma. Pigment Cell Melanoma Res..

[6] Liu H., He Z., Simon H.-U. (2013). Targeting Autophagy as a Potential Therapeutic Approach for Melanoma Therapy.

[7] Dalby K., Tekedereli I., Lopez-Berestein G., Ozpolat B. (2010). Targeting the pro-death and pro-survival functions of autophagy as novel therapeutic strategies in cancer. Autophagy.

[8] White E., DiPaola R.S. (2009). The double-edged sword of autophagy modulation in cancer. Clin. Cancer Res. Off. J. Am. Assoc. Cancer Res..

[9] Kondo Y., Kanzawa T., Sawaya R., Kondo S. (2005). The role of autophagy in cancer development and response to therapy. Nat. Rev. Cancer.

[10] Sui X., Chen R., Wang Z., Huang Z., Kong N., Zhang M., Han W., Lou F., Yang J., Zhang Q. (2013). Autophagy and chemotherapy resistance: A promising therapeutic target for cancer treatment. Cell Death Dis..

[11] Liu J.-J., Lin M., Yu J.-Y., Liu B., Bao J.-K. (2011). Targeting apoptotic and autophagic pathways for cancer therapeutics. Cancer Lett..

[12] Lai S.-L., Mustafa M.R., Wong P.-F. (2018). Panduratin a induces protective autophagy in melanoma via the ampk and mtor pathway. Phytomedicine.

[13] Mizushima N., Yoshimori T., Ohsumi Y. (2011). The role of atg proteins in autophagosome formation. Annu. Rev. Cell Dev. Biol..

[14] Levine B., Mizushima N., Virgin H.W. (2011). Autophagy in immunity and inflammation. Nature.

[15] Ndoye A., Weeraratna A.T. (2016). Autophagy-an emerging target for melanoma therapy. F1000Research.

[16] Ishaq M., Kumar S., Varinli H., Han Z.J., Rider A.E., Evans M.D., Murphy A.B., Ostrikov K. (2014). Atmospheric gas plasma–induced ros production activates tnf-ask1 pathway for the induction of melanoma cancer cell apoptosis. Mol. Biol. Cell.

[17] Keidar M., Walk R., Shashurin A., Srinivasan P., Sandler A., Dasgupta S., Ravi R., Guerrero-Preston R., Trink B. (2011). Cold plasma selectivity and the possibility of a paradigm shift in cancer therapy. Br. J. Cancer.

[18] Pasqual-Melo G., Gandhirajan R.K., Stoffels I., Bekeschus S. (2018). Targeting malignant melanoma with physical plasmas. Clin. Plas. Med..

[19] Yan D., Sherman J.H., Keidar M. (2017). Cold atmospheric plasma, a novel promising anticancer treatment modality. Oncotarget.

[20] von Woedtke T., Schmidt A., Bekeschus S., Wende K., Weltmann K.D. (2019). Plasma medicine: A field of applied redox biology. Vivo.

[21] Privat-Maldonado A., Schmidt A., Lin A., Weltmann K.D., Wende K., Bogaerts A., Bekeschus S. (2019). Ros from physical plasmas: Redox chemistry for biomedical therapy. Oxid. Med. Cell. Longev..

[22] Bekeschus S., Lin A., Fridman A., Wende K., Weltmann K.-D., Miller V. (2018). A comparison of floating-electrode dbd and kinpen jet: Plasma parameters to achieve similar growth reduction in colon cancer cells under standardized conditions. Plasma Chem. Plasma Process..

[23] Valinataj Omran A., Baitukha A., Pulpytel J., Sohbatzadeh F., Arefi-Khonsari F. (2018). Atmospheric pressure surface modification and cross-linking of uhmwpe film and inside hdpe tube by transporting discharge. Plasma Process Polym.

[24] Winter J., Brandenburg R., Weltmann K.D. (2015). Atmospheric pressure plasma jets: An overview of devices and new directions. Plasma Sources Sci. T..

[25] Kim S.J., Chung T. (2016). Cold atmospheric plasma jet-generated rons and their selective effects on normal and carcinoma cells. Sci. Rep..

[26] Bekeschus S., Rodder K., Fregin B., Otto O., Lippert M., Weltmann K.D., Wende K., Schmidt A., Gandhirajan R.K. (2017). Toxicity and immunogenicity in murine melanoma following exposure to physical plasma-derived oxidants. Oxid. Med. Cell. Longev..

[27] Zucker S.N., Zirnheld J., Bagati A., DiSanto T.M., Des Soye B., Wawrzyniak J.A., Etemadi K., Nikiforov M., Berezney R. (2012). Preferential induction of apoptotic cell death in melanoma cells as compared with normal keratinocytes using a non-thermal plasma torch. Cancer Biol..

[28] Fawcett H., Mader J.S., Robichaud M., Giacomantonio C., Hoskin D.W. (2005). Contribution of reactive oxygen species and caspase-3 to apoptosis and attenuated icam-1 expression by paclitaxel-treated mda-mb-435 breast carcinoma cells. Int. J. Oncol..

[29] Wong C.H., Iskandar K.B., Yadav S.K., Hirpara J.L., Loh T., Pervaiz S. (2010). Simultaneous induction of non-canonical autophagy and apoptosis in cancer cells by ros-dependent erk and jnk activation. PLoS ONE.

[30] Azad M.B., Chen Y., Gibson S.B. (2009). Regulation of autophagy by reactive oxygen species (ros): Implications for cancer progression and treatment. Antioxid. Redox Signal..

[31] Rafiei A., Sohbatzadeh F., Hadavi S., Bekeschus S., Alimohammadi M., Valadan R. (2020). Inhibition of murine melanoma tumor growth in vitro and in vivo using an argon-based plasma jet. Clin. Plasma Med..

[32] Sun J., Zhang X., Broderick M., Fein H. (2003). Measurement of nitric oxide production in biological systems by using griess reaction assay. Sensors.

[33] Fattahi S., Pilehchian Langroudi M., Samadani A.A., Nikbakhsh N., Asouri M., Akhavan-Niaki H. (2017). Application of unique sequence index (usi) barcode to gene expression profiling in gastric adenocarcinoma. J. Cell Commun. Signal..

[34] Fattahi S., Amirbozorgi G., Lotfi M., Navaei B.A., Kavoosian S., Asouri M., Akhavan-Niaki H. (2018). Development of a universal taqman probe for mrna gene expression analysis. Iran. J. Sci. Technol. Trans. A Sci..

[35] Livak K.J., Schmittgen T.D. (2001). Analysis of relative gene expression data using real-time quantitative pcr and the 2(-delta delta c(t)) method. Methods.

[36] Ouyang L., Shi Z., Zhao S., Wang F.T., Zhou T.T., Liu B., Bao J.K. (2012). Programmed cell death pathways in cancer: A review of apoptosis, autophagy and programmed necrosis. Cell Prolif..

[37] Saadati F., Mahdikia H., Abbaszadeh H.-A., Abdollahifar M.-A., Khoramgah M.S., Shokri B. (2018). Comparison of direct and indirect cold atmospheric-pressure plasma methods in the b 16 f 10 melanoma cancer cells treatment. Sci. Rep..

[38] Bekeschus S., Schmidt A., Niessner F., Gerling T., Weltmann K.D., Wende K. (2017). Basic research in plasma medicine—A throughput approach from liquids to cells. J. Vis. Exp..

[39] Lee H., Shon C., Kim Y., Kim S., Kim G., Kong M.G. (2009). Degradation of adhesion molecules of g361 melanoma cells by a non-thermal atmospheric pressure microplasma. New J. Phys..

[40] Gandhirajan R.K., Rodder K., Bodnar Y., Pasqual-Melo G., Emmert S., Griguer C.E., Weltmann K.D., Bekeschus S. (2018). Cytochrome c oxidase inhibition and cold plasma-derived oxidants synergize in melanoma cell death induction. Sci. Rep..

[41] Fridman G., Shereshevsky A., Jost M.M., Brooks A.D., Fridman A., Gutsol A., Vasilets V., Friedman G. (2007). Floating electrode dielectric barrier discharge plasma in air promoting apoptotic behavior in melanoma skin cancer cell lines. Plasma Chem. Plasma Process..

[42] Sagwal S.K., Pasqual-Melo G., Bodnar Y., Gandhirajan R.K., Bekeschus S. (2018). Combination of chemotherapy and physical plasma elicits melanoma cell death via upregulation of slc22a16. Cell Death Dis..

[43] Chen G., Chen Z., Wen D., Wang Z., Li H., Zeng Y., Dotti G., Wirz R.E., Gu Z. (2020). Transdermal cold atmospheric plasma-mediated immune checkpoint blockade therapy. Proc. Natl. Acad. Sci. USA.

[44] Vandamme M., Robert E., Lerondel S., Sarron V., Ries D., Dozias S., Sobilo J., Gosset D., Kieda C., Legrain B. (2012). Ros implication in a new antitumor strategy based on non-thermal plasma. Int. J. Cancer.

[45] Tanaka H., Mizuno M., Ishikawa K., Nakamura K., Kajiyama H., Kano H., Kikkawa F., Hori M. (2011). Plasma-activated medium selectively kills glioblastoma brain tumor cells by down-regulating a survival signaling molecule, akt kinase. Plasma Med..

[46] Kaushik N., Attri P., Kaushik N., Choi E. (2013). A preliminary study of the effect of dbd plasma and osmolytes on t98g brain cancer and hek non-malignant cells. Molecules.

[47] Kim S.J., Chung T., Bae S., Leem S. (2010). Induction of apoptosis in human breast cancer cells by a pulsed atmospheric pressure plasma jet. Appl. Phys. Lett..

[48] Wang M., Holmes B., Cheng X., Zhu W., Keidar M., Zhang L.G. (2013). Cold atmospheric plasma for selectively ablating metastatic breast cancer cells. PLoS ONE.

[49] Georgescu N., Lupu A.R. (2010). Tumoral and normal cells treatment with high-voltage pulsed cold atmospheric plasma jets. IEEE Trans. Plasma Sci..

[50] Ishaq M., Evans M.D., Ostrikov K.K. (2014). Atmospheric pressure gas plasma-induced colorectal cancer cell death is mediated by nox2–ask1 apoptosis pathways and oxidative stress is mitigated by srx–nrf2 anti-oxidant system. Biochim. Et Biophys. Acta (Bba)-Mol. Cell Res..

[51] Freund E., Liedtke K.R., van der Linde J., Metelmann H.R., Heidecke C.D., Partecke L.I., Bekeschus S. (2019). Physical plasma-treated saline promotes an immunogenic phenotype in ct26 colon cancer cells in vitro and in vivo. Sci. Rep..

[52] Plewa J.-M., Yousfi M., Frongia C., Eichwald O., Ducommun B., Merbahi N., Lobjois V. (2014). Low-temperature plasma-induced antiproliferative effects on multi-cellular tumor spheroids. New J. Phys..

[53] Kim J.Y., Ballato J., Foy P., Hawkins T., Wei Y., Li J., Kim S.-O. (2011). Apoptosis of lung carcinoma cells induced by a flexible optical fiber-based cold microplasma. Biosens. Bioelectron..

[54] Ja Kim S., Min Joh H., Chung T. (2013). Production of intracellular reactive oxygen species and change of cell viability induced by atmospheric pressure plasma in normal and cancer cells. Appl. Phys. Lett..

[55] Li W., Yu H., Ding D., Chen Z., Wang Y., Wang S., Li X., Keidar M., Zhang W. (2019). Cold atmospheric plasma and iron oxide-based magnetic nanoparticles for synergetic lung cancer therapy. Free Radic. Biol. Med..

[56] Barekzi N., Laroussi M. (2012). Dose-dependent killing of leukemia cells by low-temperature plasma. J. Phys. D Appl. Phys..

[57] Thiyagarajan M., Waldbeser L., Whitmill A. (2012). Thp-1 leukemia cancer treatment using a portable plasma device. Stud. Health Technol. Inform..

[58] Schmidt A., Rodder K., Hasse S., Masur K., Toups L., Lillig C.H., von Woedtke T., Wende K., Bekeschus S. (2016). Redox-regulation of activator protein 1 family members in blood cancer cell lines exposed to cold physical plasma-treated medium. Plasma Process. Polym..

[59] Bekeschus S., Wende K., Hefny M.M., Rodder K., Jablonowski H., Schmidt A., Woedtke T.V., Weltmann K.D., Benedikt J. (2017). Oxygen atoms are critical in rendering thp-1 leukaemia cells susceptible to cold physical plasma-induced apoptosis. Sci. Rep..

[60] Thiyagarajan M., Anderson H., Gonzales X.F. (2014). Induction of apoptosis in human myeloid leukemia cells by remote exposure of resistive barrier cold plasma. Biotechnol. Bioeng..

[61] Zhang X., Li M., Zhou R., Feng K., Yang S. (2008). Ablation of liver cancer cells in vitro by a plasma needle. Appl. Phys. Lett..

[62] Zhao S., Xiong Z., Mao X., Meng D., Lei Q., Li Y., Deng P., Chen M., Tu M., Lu X. (2013). Atmospheric pressure room temperature plasma jets facilitate oxidative and nitrative stress and lead to endoplasmic reticulum stress dependent apoptosis in hepg2 cells. PLoS ONE.

[63] Tan X., Zhao S., Lei Q., Lu X., He G., Ostrikov K. (2014). Single-cell-precision microplasma-induced cancer cell apoptosis. PLoS ONE.

[64] Guerrero-Preston R., Ogawa T., Uemura M., Shumulinsky G., Valle B.L., Pirini F., Ravi R., Sidransky D., Keidar M., Trink B. (2014). Cold atmospheric plasma treatment selectively targets head and neck squamous cell carcinoma cells. Int. J. Mol. Med..

[65] Kang S., Cho J., Chang J., Shin Y., Kim K., Park J., Yang S., Lee J., Moon E., Lee K. (2014). Nonthermal plasma induces head and neck cancer cell death: The potential involvement of mitogen-activated protein kinase-dependent mitochondrial reactive oxygen species. Cell Death Dis..

[66] Hasse S., Seebauer C., Wende K., Schmidt A., Metelmann H.R., von Woedtke T., Bekeschus S. (2019). Cold argon plasma as adjuvant tumour therapy on progressive head and neck cancer: A preclinical study. Appl. Sci..

[67] Reuter S., Tresp H., Wende K., Hammer M.U., Winter J., Masur K., Schmidt-Bleker A., Weltmann K.-D. (2012). From rons to ros: Tailoring plasma jet treatment of skin cells. IEEE Trans. Plasma Sci..

[68] Han X., Klas M., Liu Y., Sharon Stack M., Ptasinska S. (2013). DNA damage in oral cancer cells induced by nitrogen atmospheric pressure plasma jets. Appl. Phys. Lett..

[69] Panngom K., Baik K.Y., Nam M.-K., Han J., Rhim H., Choi E. (2013). Preferential killing of human lung cancer cell lines with mitochondrial dysfunction by nonthermal dielectric barrier discharge plasma. Cell Death Dis..

[70] Galluzzi L., Vitale I., Aaronson S.A., Abrams J.M., Adam D., Agostinis P., Alnemri E.S., Altucci L., Amelio I., Andrews D.W. (2018). Molecular mechanisms of cell death: Recommendations of the nomenclature committee on cell death 2018. Cell Death Differ.

[71] Helfinger V., Schroder K. (2018). Redox control in cancer development and progression. Mol. Asp. Med..

[72] Gotoh T., Mori M. (1999). Arginase ii downregulates nitric oxide (no) production and prevents no-mediated apoptosis in murine macrophage-derived raw 264.7 cells. J. Cell Biol..

[73] Mohana-Kumaran N., Hill D.S., Allen J.D., Haass N.K. (2014). Targeting the intrinsic apoptosis pathway as a strategy for melanoma therapy. Pigment Cell Melanoma Res..

[74] Raisova M., Hossini A.M., Eberle J., Riebeling C., Wieder T., Sturm I., Daniel P.T., Orfanos C.E., Geilen C.C. (2001). The bax/bcl-2 ratio determines the susceptibility of human melanoma cells to cd95/fas-mediated apoptosis. J. Investig. Dermatol..

[75] Yazdani Z., Mehrabanjoubani P., Biparva P., Rafiei A. (2020). Cytotoxicity Effect of Cold Atmospheric Plasma on Melanoma (B16-F10), Breast (MCF-7) and Lung (A549) Cancer Cell Lines Compared with Normal Cells. J. Mazandaran Univ. Med. Sci..

[76] Turrini E., Laurita R., Stancampiano A., Catanzaro E., Calcabrini C., Maffei F., Gherardi M., Colombo V., Fimognari C. (2017). Cold atmospheric plasma induces apoptosis and oxidative stress pathway regulation in t-lymphoblastoid leukemia cells. Oxidative Med. Cell. Longev..

[77] Mirpour S., Piroozmand S., Soleimani N., Faharani N.J., Ghomi H., Eskandari H.F., Sharifi A.M., Mirpour S., Eftekhari M., Nikkhah M. (2016). Utilizing the micron sized non-thermal atmospheric pressure plasma inside the animal body for the tumor treatment application. Sci. Rep..

[78] Bekeschus S., Clemen R., Nießner F., Sagwal S.K., Freund E., Schmidt A. (2020). Medical gas plasma jet technology targets murine melanoma in an immunogenic fashion. Adv. Sci..

[79] Kondapuram S.K., Sarvagalla S., Coumar M.S. (2019). Targeting autophagy with small molecules for cancer therapy. J. Cancer Metastasis Treat.

[80] Russo M., Russo G.L. (2018). Autophagy inducers in cancer. Biochem. Pharmacol..

[81] Booth L.A., Tavallai S., Hamed H.A., Cruickshanks N., Dent P. (2014). The role of cell signalling in the crosstalk between autophagy and apoptosis. Cell. Signal..

[82] Shen S., Zhang Y., Zhang R., Tu X., Gong X. (2014). Ursolic acid induces autophagy in u87mg cells via ros-dependent endoplasmic reticulum stress. Chem.-Biol. Interact..

[83] Brulle L., Vandamme M., Ries D., Martel E., Robert E., Lerondel S., Trichet V., Richard S., Pouvesle J.M., Le Pape A. (2012). Effects of a non thermal plasma treatment alone or in combination with gemcitabine in a mia paca2-luc orthotopic pancreatic carcinoma model. PLoS ONE.

[84] Palumbo S., Pirtoli L., Tini P., Cevenini G., Calderaro F., Toscano M., Miracco C., Comincini S. (2012). Different involvement of autophagy in human malignant glioma cell lines undergoing irradiation and temozolomide combined treatments. J. Cell. Biochem..

